# Sememe Heredity of Action Semantics: Evidence From the Priming Effect and Prospective Memory

**DOI:** 10.3389/fpsyg.2020.02057

**Published:** 2020-09-25

**Authors:** Zhanyu Yu, Yue Ma, Lijuan Wang

**Affiliations:** ^1^School of Education Science, Jiangsu Normal University, Xuzhou, China; ^2^Mental Health Education Center for College Students, Jiangsu Normal University, Xuzhou, China; ^3^School of Psychology, Northeast Normal University, Changchun, China

**Keywords:** action semantics, sememe heredity, enactment effect, verb semantics, related association

## Abstract

The sememe heredity of action semantics may be affected by the related association of a verb or noun in an action phrase and the related association between one action phrase and another. The motor encoding theory and the five-component view of the subject-performed task supported by the verb’s specificity highly promote the enactment effect. However, the episodic integration theory emphasizes the role of semantic integration between the verb and noun on the enactment effect. In this study, a subject-performed task was combined with a priming paradigm and found that verb-semantic priming quantity was more significant than that of noun-semantic priming quantity under motor encoding in Experiment 1. Besides, it was observed that the verb-semantic association might play a more significant role in the sememe heredity of action semantics. Therefore, in Experiment 2, a subject-performed task was combined with the dual task of prospective memory. Results showed that the accuracy of prospective memory targets related to the learning phrases was significantly higher compared to that of the prospective memory targets unrelated to the learning phrases. Besides, the above difference is more evident verbally compared to motor encoding conditions. Thus, the sememe heredity of action semantics may rely on the related association of action semantic contents rather than on the semantic processing form of external motor encoding.

## Introduction

The theory of “lexical rules” proposed by Leech (1983) holds that sememe heredity contributes to the dynamic evolution of polysemy structure of words. For example:

The word “milk” can be defined in two different ways.

Definition 1: A white liquid produced by female mammals.

Definition 2: A whitish liquid or juice obtained from certain plants and trees.

Definition 1 and 2 share the two sememes “white” and “liquid.” Therefore, this is the sememe heredity process based on similarity and related association, which can be compared to biological or genetic inheritance (Lv and Dai, 2000). The related association refers to a situation whereby individuals express a similar feeling to the current thing and recall another similar thing with graphics, schema, semantics, structure, and method (Zhang, 1997). Notably, Zhang (2003) concluded that the theory of the lexical rules could explain the polysemous structure of nouns, and this association is the key to sememe heredity. However, besides the processing of noun-semantic information, people commonly use or identify action-output information in daily life. Also, individuals tend to change the environment through actions to achieve specific goals (Zimmer and Cohen, 2001). For example, if someone asks, “what did you do today?” your answer should include some action phrases such as eating hamburgers or playing games. Therefore, studying the semantic information of action phrases that are action semantics exhibit more application value.

For example, one can ask whether action semantics are equally suitable for the “lexical rules” theory or whether association plays a vital role in the sememe heredity of action semantics. Unlike noun semantics, action semantics are combined; that is, they involve verbs and nouns (Liu and Wang, 2018). Therefore, distinguishing their respective roles is critical in answering the above questions. Similarly, the association should be grouped into a verb and noun association. However, linguists have not focused on the problem due to sememe heredity of action semantics. The theoretical basis and research conclusions in the field of memory provide inadequate evidence on the role of verbs and nouns in action phrases, as will be discussed below.

### Theoretical Interpretation of Differences in Verb and Noun Processing in Action Memory

Action memory refers to conscious memory activity accompanied by manipulated actions (Yu and Wang, 2017; Li and Wang, 2018). “Subject performed task” (SPT) is the standard paradigm for studying action memory. Subjects are asked at random to complete an SPT by performing simple tasks (such as breaking a pencil or opening a book) or to complete a verbal task (VT) by reading or listening to a description of the same task without enactment (Cohen, 1981; Leynes, and Kakadia, 2013). The memory performance is superior in SPT or motor encoding compared with VT or verbal encoding, and it is known as the enactment effect (Nilsson, 2000; Peterson and Mulligan, 2010; Zhang and Zuber, 2020).

Some theories have proposed differences in the specific processing of verbs and nouns on the enactment effect. Notably, the motor encoding theory particularly broadens the dual-code theory of Paivio (1969) by proposing an independent motor-encoding process (Engelkamp and Zimmer, 1985). It highlights that motor encoding can enhance item-specific information whereby individuals specifically pay attention to information related to the movement component of an action (Spranger et al., 2008). The structural processing viewpoint of motor encoding categorizes a “subject-performed task” into five components, including lexical-semantic processing, the formation of volition, movement and motor programming, motor execution and monitoring, and motor evaluation. Moreover, this theory holds that motor encoding makes individuals pay more attention to the action components of phrases. Eventually, information about the movement component of the action represented in verbs than nouns (Hunt and McDaniel, 1993) becomes obvious (Zimmer, 2001). Briefly, both theories support the view that the specificity of verb items, which refers to the characteristics and attributes of verbs, makes them unique and distinguishes them from nouns or other items (Kubik et al., 2016) thereby highly contributing to the enactment effect.

Furthermore, episodic integration theory emphasizes the roles of semantic and episodic integration on the enactment effect. Episodic integration combines operators’ actions with objects, while semantic integration combines verbs with nouns (Kormi-Nouri and Nilsson, 1998; Wang and Li, 2014). This theory holds that motor encoding can integrate verbs and nouns into a compact memory unit. Moreover, the integrating mechanism for the two improves item specificity and memory advantage, which occurs under the motor encoding condition. Besides, the theory does not emphasize the difference between the contributions of the verb and noun specificity to the enactment effect, but rather it integrates the two (Kormi-Nouri, 1995; Kormi-Nouri and Nilsson, 2001).

### Empirical Study on the Differences in Verb and Noun Processing in Action Memory

Like the previous contrasting theoretical viewpoints, empirical studies have not achieved a consistent conclusion. Denis et al. (1991) found that motor encoding only improved the cued recall performance of verbs. Besides, verb-semantic similar actions greatly affect the target actions compared to noun-semantic similar actions (Engelkamp and Zimmer, 1994). Both studies suggest that verb-item specificity contributes highly to the enactment effect. Other studies have suggested that the specificities of the verb and noun items contribute equally to the enactment effect. Motor encoding exhibits a similar promoting effect on the free recall scores (Earles and Kersten, 2000) and recognition scores (Steffens et al., 2006) of verbs and nouns.

However, all these previous studies have limitations. The free recall results in the study by Earles and Kersten (2000) have a floor effect. Besides, Steffens et al. (2006) used free recall, cued recall, and recognition in turn in their research, and their results were potentially influenced by the order and fatigue effect (Li et al., 2016). The order effect, in this case, refers to the deviation of results caused by the fixed order of stimulus or tests (Mantonakis et al., 2009). Besides, the fatigue effect refers to the deviation of results caused by the consumption of attentional resources and is accompanied by subjective fatigue after long-term cognitive processing (Lorist, 2008). In the study by Engelkamp and Zimmer (1994), the subjects probably did not process interference materials at the retrieval stage. Therefore, motor encoding may not have been affected (Mohr et al., 1989). A study by Denis et al. (1991) used cued recall; however, this method does not exclude the negative influence of relational information.

Notably, there are more semantic nodes of nouns corresponding to a verb than semantic nodes of verbs corresponding to a noun (Kersten and Billman, 1997; Imai and Okada, 2005). Moreover, nouns have more related structures and are easier to remember, than verbs (Childers and Tomasello, 2006). Other studies suggest that verbs and nouns are not similar at the semantic organization level; that is, nouns are hierarchically organized while verbs are matrix organized. The term “matrix organization” implies that there is no interdependence between attributes and their organization is conducted separately (Parisien et al., 2019). A specific noun can relate to other nouns; however, the connection of one verb with others is difficult to identify (Earles and Kersten, 2000). Higher relational processing may make it easier for subjects to recall verbs, thereby justifying that motor encoding improves the specificity of verb items. Besides, the relational processing of verbs and nouns in uniqueness action phrases such as “knead the dough” is higher than that in non-uniqueness action phrases such as “tie the laces.” Therefore, in this study, the free recall rather than cued recall performance of non-uniqueness action phrases was tested in Experiment 1 to minimize the deviation resulting from relational processing.

Furthermore, motor encoding theory and the structural processing viewpoint of motor encoding emphasize the importance of verb specificity (Zimmer, 2001; Spranger et al., 2008). However, from the memory retrieval perspective, empirical research in this field uses the recognition, cued recall, and free recall to indirectly explore how verb and noun specificity impacts the enactment effect (Earles and Kersten, 2000; Steffens et al., 2006). The differences between encoding and retrieval of action memory may be associated with the contradictory conclusions from existing studies (Mohr et al., 1989; Nyberg et al., 2001). Therefore, the advantage of the priming paradigm is to directly explore cognitive processing from the encoding stage (Collins and Loftus, 1975; Wang and Wang, 2008). Precisely, the reaction time of verb (or noun)-priming phrases are shorter than those of noun (or verb)-priming phrases if the specificity of verbs (or nouns) highly contributes to the enactment effect. Therefore, Experiment 1 examined the different roles of the verb- and noun-semantic association in the sememe heredity of action semantics by combining subject-performed tasks with a priming paradigm.

Sememe heredity is based on association. The association of action semantics is reflected in the verb-noun association and the association of target action phrases (hit the nail) with related action phrases (wave the hammer) (Zimmer, 2001). The dual-task of prospective memory may provide a new perspective on the impact of the association of target-related action phrases on sememe heredity of action semantics. Notably, prospective memory implies the ability to remember to initiate and execute an intended action in the future. The standard dual-task includes prospective memory tasks and an ongoing task (Guynn, 2008; Haas et al., 2020). The prospective memory instructions of action events are action phrases such as “breaking a pencil” or “opening a book”; The ongoing task is to sort the words into regular items by pressing keys. The prospective memory is embedded in an ongoing task (Li and Wang, 2015). The prospective memory instructions are in a special “to be executed” target state (McDaniel et al., 1998). Similarly, the related action phrases during motor encoding may be in a state of “to be executed” (Zimmer, 2001). Notably, motor encoding may improve prospective memory accuracy if the related action phrases are regarded as the target instructions of prospective memory. Therefore, in Experiment 2, we examined whether the related association contributes to the sememe heredity of action semantics by combining subject-performed tasks with the dual task of prospective memory.

## Experiment 1

This experiment combined a subject-performed task with a priming paradigm to explore the different roles of the verb- and noun-semantic association in the sememe heredity of action semantics. We assumed that a reliable enactment effect would be obtained, and a verb-semantic priming quantity would be more significant than a noun-semantic priming quantity under motor encoding.

### Methods

#### Design

The experiment used a 2 × 3 (type of encoding: SPT vs. VT × relationship between priming and target action phrases: same nouns vs. same verbs vs. unrelated) mixed factorial design in which the former factor varied between subjects and the latter within subjects. The dependent variable was the free recall scores. Correct recall of the phrase was scored as 1 point despite the discrepancies in expression. Failure to recall the phrase and incorrectly recall the verb or noun were scored as 0 (Liu and Wang, 2018). The semantic priming quantity of the noun or verb was the second dependent variable. This refers to the lexical decision time (reaction time) for action phrases unrelated to the priming phrases minus the lexical decision time of action phrases whose nouns or verbs were identical to the priming phrases.

#### Subjects

Before the experiment, G^∗^Power (Erdfelder et al., 1996) was used to calculate the sample size. Compared with the study of Li and Wang (2018), a six-level mixed factorial design was adopted and expected an effect size of 0.2, a 0.05 probability error, a power of 0.9, and a correlation among repeated measures of 0.5. The sample size was 36 subjects. Notably, 40 subjects composed of 22 males and 18 females (*M*_*age*_ = 19.52 years, *SD*_*age*_ = 0.48 years) were randomly assigned in equal numbers to either the SPT or the VT encoding condition of the formal experiment. All the subjects were right-handed, their eyesight (or corrected visual acuity) was at least 1.0, and none had previously participated in a similar experiment. The groups did not differ significantly in age, *F*(1, 38) = 0.067, *p* = 0.797, η^2^*_*p*_* = 0.002, or in years of formal education, *F*(1, 38) = 0.218, *p* = 0.643, η^2^*_*p*_* = 0.006.

#### Materials

Another group of 40 subjects on 120 action phrases were assessed as follows:

(1) Collocation degree evaluation: They judged whether the collocations of verbs and nouns in the action phrases are reasonable, using a seven-point scale evaluation.

(2) Familiarity assessment: They judged whether the subjects knew how to operate the verbs in the action phrases using a seven-point scale assessment. Besides, it was determined whether they knew what the nouns in the phrases represented using a seven-point scale assessment.

(3) Structural or functional operational representation evaluation: They judged whether the operation of the action phrases involved the use function of the object, or only grasped and then generated displacement on the object (Rizzolatti and Matelli, 2003; Rueschemeyer et al., 2010). Structural operation refers to the cognitive processing of “picking up and moving objects,” the characters of shape and size are processed, for example, “tear the stamp.” Functional operation refers to the function of objects and is mainly responsible for processing the use of objects such as “turn the steering wheel” (Bub et al., 2008; Buxbaum and Kalénine, 2010).

(4) Semantic evaluation: They judged whether the association of priming with the target phrases such that they exhibit a similar noun, but the verb operates differently (e.g., look in a mirror vs. throw a mirror), or the verb is the same, but the nouns are not from the same semantic category (e.g., toss the coin vs. throw the embroidered ball), or are unrelated (e.g., fly a kite vs. close the window).

(5) Uniqueness evaluation: They judged whether one collocation existed between the verbs and nouns in the action phrases.

After the evaluation, 9 action phrases with poor collocation degrees, 2 unfamiliar phrases with verbs, 8 unfamiliar phrases with nouns, and 11 unique phrases were deleted, leaving 73 valid phrases. Literature analysis revealed that 32–48 materials had been adopted in the field of action memory since 2000 (Earles and Kersten, 2002; Peterson and Mulligan, 2010). In this study, 58 action phrases were selected, out of which 10 phrases were used for structural or functional operational representation judgment exercises. The remaining 48 phrases were used in the formal experiment, out of which 24 were priming phrases, and 24 were target phrases. The priming phrases were divided into 3 equal categories: One with nouns similar to the target phrases, one with verbs similar to the target phrases, and one with neither nouns nor verbs similar to the target phrases. The structural and functional operational representation phrases each accounted for half of the phrases. Each phrase included a verb and a noun, and all experimental materials composed of 3 or 4 Chinese characters. The materials did not involve phrases related to body parts or the objects in the laboratory (Steffens et al., 2007).

Repeated measurement analysis of variance showed no significant difference in collocation degree between the priming phrases (*M* = 6.998, *SD* = 0.007) and the target phrases (*M* = 6.996, *SD* = 0.01), *F*(1, 23) = 0.657, *p* = 0.426, η^2^*_*p*_* = 0.028. Besides, no significant difference was observed in verb familiarity between the priming phrases (*M* = 6.997, *SD* = 0.008) and the target phrases (*M* = 6.994, *SD* = 0.011), *F*(1, 23) = 1, *p* = 0.328, η^2^*_*p*_* = 0.042. No significant difference was observed in noun familiarity between the priming phrases (*M* = 6.993, *SD* = 0.012) and the target phrases (*M* = 6.997, *SD* = 0.008), *F*(1, 23) = 1.643, *p* = 0.213, η^2^*_*p*_* = 0.067. However, the structural (*M* = 1.019, *SD* = 0.03) and functional (*M* = 1.981, *SD* = 0.026) performances of the priming phrases showed significant differences: *F*(1, 11) = 10297.737, *p* < 0.001, η^2^*_*p*_* = 0.999. Moreover, the structural (*M* = 1.01, *SD* = 0.025) and functional (*M* = 1.992, *SD* = 0.022) performance evaluation results of the target phrases were significantly different, *F*(1, 11) = 12514.108, *p* <0.001, η^2^*_*p*_* = 0.999.

#### Procedure

Herein, each subject was tested individually. The experiment was divided into three stages: Exercise, study, and test. The procedures for the first two stages were compiled using E-prime 2.0. In the exercise stage, the subjects performed structural (J key with middle finger) or functional (G key with index finger) operation representation judgment feedback exercises on 10 action phrases. The exercises were repeated until 100% accuracy was achieved.

**FIGURE 1:**
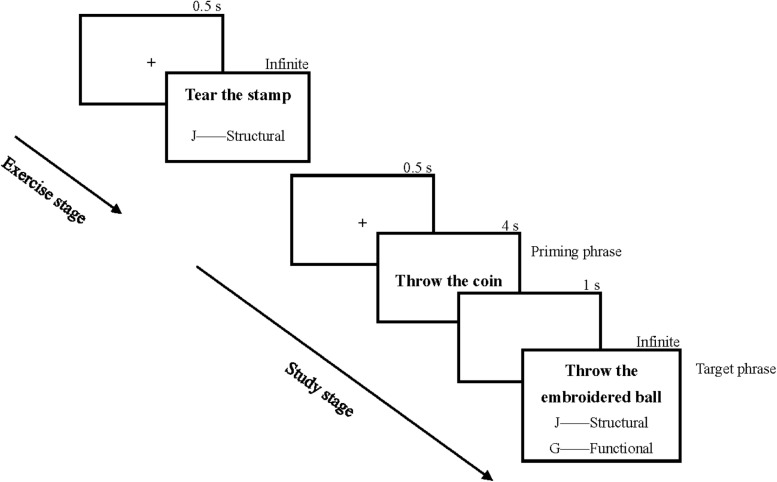
Exercise and study stages used in Experiment 1.

In the study stage, a “+” fixation appeared at the center of the screen, and then two action phrases were presented in sequence. The subjects needed to remember the first priming phrase by motor or verbal encoding and then complete the second target phrase’s operational representation judgment task. A one-second interval was set between the priming phrase and the target phrase. The subjects specifically used their left hand or arm, pretending to perform the first phrase (the priming phrase) without objects in the motor encoding group. The subjects read the priming phrases silently without objects or hand/arm movements in the verbal encoding group. In the operational representation judgment tasks, they needed to make a judgment while ensuring correctness quickly. The time setting for the procedure is shown in Figure 1.

In the free recall stage, the subjects were asked to fill in a personal information questionnaire so that the learning content was not kept in working memory. They were then required to freely recall the priming phrases in no specific order and write the recalled phrases, verbs, and nouns on paper. After the experiment, each of the subjects was given a beautiful gift. The entire experiment lasted about 15 min.

### Results

#### Free Recall

One-way ANOVA analysis showed that the main effect of the type of encoding was significant, *F*(1, 39) = 22.589, *p* < 0.001, η^2^*_*p*_* = 0.373. This suggested that free recall performance under motor encoding was significantly higher than under verbal encoding, implying an enactment effect.

#### Semantic Priming Quantity

Repeated measurement analysis of variance showed that the main effect of type of encoding was significant, *F*(1, 38) = 4.254, *p* < 0.05, η^2^*_*p*_* = 0.101, meaning, the semantic priming quantity under motor encoding (*M* = 437.222, *SD* = 424.905) was significantly larger than that under verbal encoding (*M* = 203.019, *SD* = 278.073). The main effect of semantic priming type was significant, *F*(1, 38) = 4.344, *p* < 0.05, η^2^*_*p*_* = 0.103, implying that the verb-semantic priming quantity (*M* = 393.609, *SD* = 555.333) was significantly greater than the noun-semantic priming quantity (*M* = 246.631, *SD* = 208.295). Besides, the interaction between the type of encoding and semantic priming type was significant, *F*(1, 38) = 4.185, *p* < 0.05, η^2^*_*p*_* = 0.099.

**TABLE 1:** Mean proportion of free recall under SPT and VT conditions, and mean proportion and reaction time across the type of encoding, and the relationship between priming phrases and target phrases.

Type of encoding	Free recall		Relationship between priming phrases and target phrases											
			Nouns are the same				Verbs are the same				Unrelated			
			Accuracy		Reaction time (ms)		Accuracy		Reaction time (ms)		Accuracy		Reaction time (ms)	
	*M*	*SD*	*M*	*SD*	*M*	*SD*	*M*	*SD*	*M*	*SD*	*M*	*SD*	*M*	*SD*
Motor encoding	0.61	0.21	0.86	0.12	2177.83	482.41	0.82	0.1	1906.59	735.69	0.83	0.12	2469.43	534.18
Verbal encoding	0.36	0.11	0.88	0.13	1666.67	502.66	0.86	0.1	1653.94	491.73	0.89	0.15	1858.33	642.21

**FIGURE 2:**
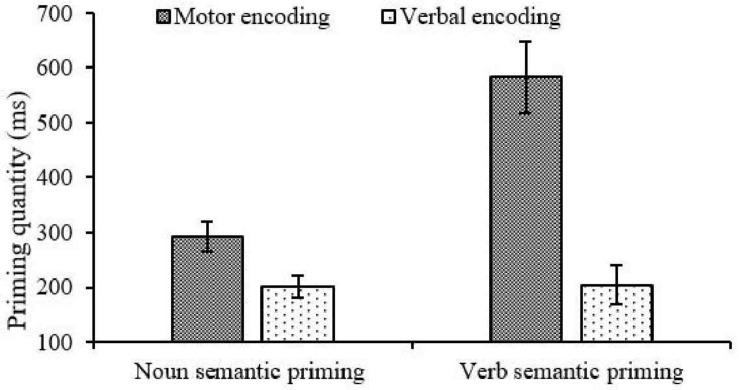
Semantic priming quantity across “type of encoding” and “type of semantic priming.”

Further, simple effect analysis showed that the type of encoding made a significant difference to the verb-semantic priming quantity, *F*(1, 38) = 5.037, *p*<0.05, η^2^*_*p*_* = 0.119, this indicated that the verb-semantic priming quantity (*M* = 582.838, *SD* = 655.154) under the motor encoding condition was significantly greater than under verbal encoding (*M* = 204.381, *SD* = 358.342). Besides, no significant difference was observed in noun-semantic priming quantity for type of encoding, *F*(1, 38) = 1.031, *p* = 0.316, η^2^*_*p*_* = 0.026 (motor: *M* = 291.606, *SD* = 269.297; verbal: *M* = 201.656, *SD* = 290.665; see Figure 2). Taking the accuracy as an index, the above main effects and interactions of noun- and verb-semantic priming quantities were not significant, *p*_*min*_ = 0.12, η^2^*p*_*max*_ = 0.063.

### Discussion

Previous studies indicate that the recognition scores of verbs (Engelkamp et al., 1995), the free recall scores of verbs (Freeman and Ellis, 2003), and the free recall and cued recall of word pairs (Engelkamp et al., 1990) are significantly higher under the motor encoding condition than under the verbal encoding condition. In this study, the priming paradigm was used to bridge the gap left by previous studies that only explored the impact of verb specificity on the enactment effect from the memory retrieval perspective. Similarly, verb-semantic priming quantity was found to be more significant under the motor encoding condition, and verb-specific processing highly contributed to the enactment effect. Therefore, it can be inferred that verb-semantic association plays a more significant role than noun-semantic association in the sememe heredity of action semantics.

## Experiment 2

The semantic spreading activation model holds that memory is a network structure composed of many interconnected nodes representing concepts (Collins and Loftus, 1975; Gao et al., 2008). The semantic node of a concept is activated when a concept is processed. The semantic connections of related concepts are activated and spread through the network, directly to connected nodes, and then spread to other nodes located farther away (Kopp et al., 2013). Following this model, we can infer that the association of action semantics is manifested in verb and noun semantics within and between action phrases.

Literature reports reveal that semantic processing under the motor encoding condition focuses on action phrases, which activate representations related to actions (Zimmer, 2001). However, individuals do not have to perform all the actions highlighted in the brain, but only the target actions. After that, they are registered as “executed,” whereas actions activated but not performed create a target group of actions “to be executed” under certain circumstances—a task list—which can be regarded as prospective memory (Zimmer, 2001). Similarly, the automatic processing theory of prospective memory holds that when individuals form a prospective memory connection between target clues and execution intentions, the connection is in a special subliminal “to be executed” target state. However, the individuals will also engage in ongoing tasks (OT) that interfere with it (McDaniel et al., 1998; Haas et al., 2020).

Therefore, it can be assumed that the prospective memory accuracy of prospective memory targets related to learning phrases should be significantly higher than that of prospective memory targets not related to learning phrases if the related association contributes to the sememe heredity of action semantics. Moreover, the relational processing level of motor encoding is lower than that of verbal encoding (Golly-Häring and Engelkamp, 2003; von Essen, 2005; Li et al., 2016). The above difference may be more evident under the verbal encoding condition. These hypotheses were examined in Experiment 2 by combining the subject-performed task with the dual task of prospective memory.

### Methods

#### Design

The experiment used a 2 × 2 (type of encoding: SPT vs. VT × relationship between learning phrases and prospective memory targets: related vs. unrelated) between-subject design. The dependent variables included free recall scores, prospective memory accuracy, ongoing task accuracy, and ongoing task reaction time.

#### Subjects

Due to the between-subjects design, we expected an effect size of 0.3 (Li and Wang, 2018), a power of 0.9, and a correlation among repeated measures of 0.5. The sample size was 76 subjects. A total of 80 subjects were recruited; 35 males and 45 females (*M*_*age*_ = 19.56 years, *SD*_*age*_ = 0.56 years), and they were randomly and equally divided into four groups. The groups did not differ significantly in age, *F*(1, 76) = 0.206, *p* = 0.651, η*^2^_*p*_* = 0.003, or in years of formal education, *F*(1, 76) = 0.384, *p* = 0.537, η^2^*_*p*_* = 0.005. The other standards used were similar to those in Experiment 1.

#### Materials

Another group of 40 subjects were tested on 159 action phrases. Results showed that collocation degree, familiarity, and structural or functional operational representation were identical to those in Experiment 1. In the semantic evaluation, subjects were required to judge the degrees between 3 learning phrases and 3 related or unrelated prospective memory targets. A seven-point scale was used to evaluate the results. From this process, 35 phrases with poor collocation degrees, 3 unfamiliar phrases with verbs, and 7 unfamiliar phrases with nouns were excluded, leaving 119 valid phrases.

The formal experimental materials consisted of 109 action phrases. The 3 learning phrases included “cut the potato,” “light the candle,” and “hit the nail.” The 3 prospective memory targets related to learning phrases were “hold the chicken knife,” “strike the match,” and “wave the hammer.” The 3 prospective memory targets unrelated to learning phrases were “paste the couplets,” “drip the eyedrops,” and “swipe the transportation card.” Notably, each structural and functional operational representation phrase accounted for half of the remaining 100 phrases; 20 were used in the exercise stage while 80 were used for ongoing tasks. The selection criteria for the materials were the same as those in Experiment 1.

**TABLE 2:** Mean proportion of free recall under SPT and VT conditions, and mean proportion of prospective memory across type of encoding, and the relationship between learning phrases and prospective memory targets.

Type of encoding	Relationship between learning phrases and prospective memory targets															
	Related								Unrelated							
	Free recall		PM accuracy		OT accuracy		OT reaction time (ms)		Free recall		PM accuracy		OT accuracy		OT reaction time (ms)	
	*M*	*SD*	*M*	*SD*	*M*	*SD*	*M*	*SD*	*M*	*SD*	*M*	*SD*	*M*	*SD*	*M*	*SD*
Motor encoding	0.78	0.31	0.65	0.3	0.92	0.04	1860.88	415.6	0.65	0.39	0.47	0.27	0.91	0.08	1668.78	352.34
Verbal encoding	0.48	0.35	0.8	0.2	0.91	0.08	1767.4	524.83	0.46	0.37	0.37	0.28	0.91	0.05	1719.1	563.16

*PM, prospective memory; OT, ongoing task.*

Moreover, repeated measurement analysis of variance showed that the structural (*M* = 1.014, *SD* = 0.029) and functional (*M* = 1.98, *SD* = 0.035) performance of action phrases in the ongoing task were significantly different, *F*(1, 39) = 17508.363, *p* < 0.001, η^2^*_*p*_* = 0.998. The judgment scores of the subjects on prospective memory targets related to learning phrases (*M* = 6.999, *SD* = 0.006) were significantly higher than that for prospective memory targets unrelated to learning phrases (*M* = 1.001, *SD* = 0.005), *F*(1, 49) = 31333872.111, *p* < 0.001, η^2^*_*p*_* = 1.

#### Procedure

**FIGURE 3:**
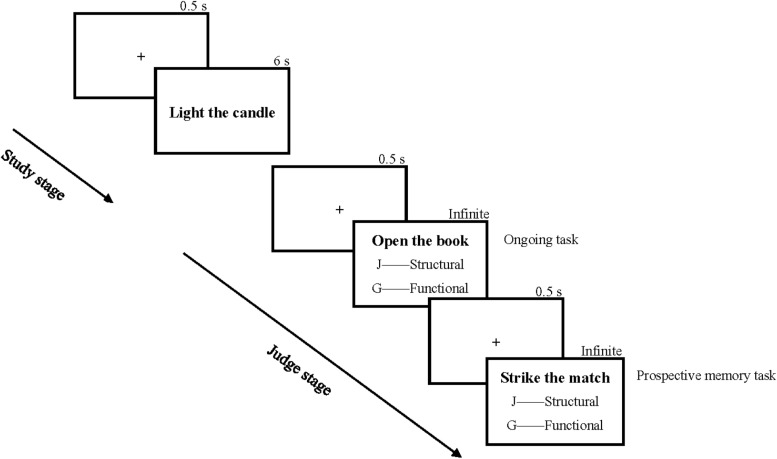
Study and judge stages used in Experiment 2.

Herein, each subject was tested individually. The experiment was divided into 4 stages: exercise, study, judge, and test. The procedures in the first three stages were compiled using E-prime 2.0. The exercise stage is as highlighted in Experiment 1. In the study stage, firstly, the subjects were instructed to learn 3 action phrases under motor or verbal encoding conditions. Each phrase was presented with a “+” fixation as an interval. Then, the subjects were required to complete the ongoing task embedded in prospective memory phrases that were categorized into related or unrelated to learning phrases. The time setting of the procedure is shown in Figure 3. In the judge stage, the subjects were instructed to finish the ongoing task, that is, they made structural or functional operational judgments fast enough while ensuring correctness. However, they were to stop and press the space key at the moment they saw any of the 3 prospective memory targets.

In the free recall stage, the subjects were asked to provide personal information via a questionnaire. They were allowed to freely recall the prospective memory targets in no specific order as in Experiment 1. After completion of the task, each subject was given a beautiful gift. The entire experiment lasted about 20 min.

### Results

#### Free Recall

One-way ANOVA analysis showed that the main effect of type of encoding was significant, *F*(1, 76) = 9.708, *p* < 0.01, η^2^*_*p*_* = 0.113, suggesting that free recall performance under motor encoding (*M* = 0.717, *SD* = 0.357) was significantly better than under verbal encoding (*M* = 0.467, *SD* = 0.359); that is, there was an enactment effect.

#### PM Accuracy

Two-way ANOVA analysis showed that the main effect of type of encoding was not significant, *F*(1, 76) = 1.177, *p* = 0.675, η^2^*_*p*_* = 0.002, implying that the accuracy of prospective memory under motor and verbal encodings was not significantly different. The main effect of the relationship between learning phrases and prospective memory targets was significant, *F*(1, 76) = 26.899, *p* < 0.001, η^2^*_*p*_* = 0.261, showing that the accuracy of prospective memory targets related to learning phrases (*M* = 0.725, *SD* = 0.26) was significantly higher than that of prospective memory targets unrelated to learning phrases (*M* = 0.417, *SD* = 0.28). Similarly, interaction between them was significant, *F*(1, 76) = 4.421, *p* < 0.05, η^2^*_*p*_* = 0.055.

**FIGURE 4:**
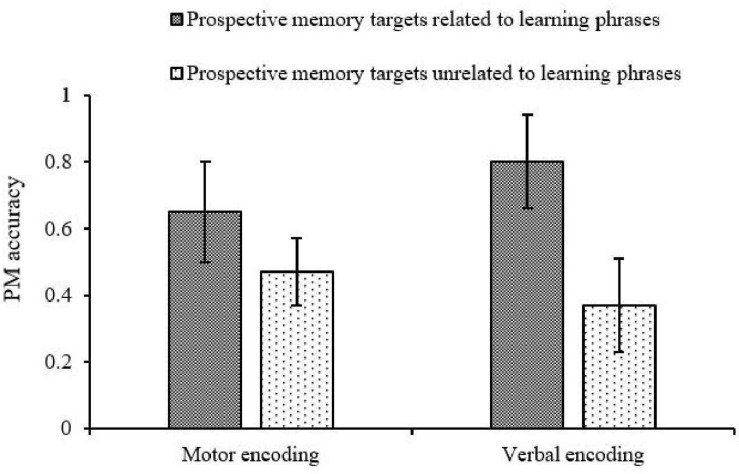
PM accuracy across “type of encoding” and “the relationship between learning phrases and prospective memory targets.”

Further simple effect analysis showed that under the motor encoding condition, the accuracy of prospective memory targets related to learning phrases was significantly higher than that of prospective memory targets unrelated to learning phrases, *F*(1, 38) = 4.142, *p* < 0.05, η^2^*_*p*_* = 0.098. However, under the verbal encoding condition, the accuracy of prospective memory targets related to learning phrases remained markedly higher than that of prospective memory targets unrelated to learning phrases [*F*(1, 38) = 31.175, *p* < 0.001, η^2^*_*p*_* = 0.451; see Figure 4]. The main effects and interactions of accuracy and reaction time in the ongoing task were not significant, *p*_*min*_ = 0.26, η^2^*p*_*max*_ = 0.017.

### Discussion

Previous studies consider motor encoding as a memory strategy which improves subjects’ prospective memory (Schaefer et al., 1998; Pereira et al., 2012a), or modifies the effect of age on prospective memory (Passolunghi et al., 1995; Pereira et al., 2012b; Li and Wang, 2015). By contrary, this study used the prospective memory paradigm as a research method to reveal the structural processing of motor encoding. Results showed that the relational processing level between learning phrases and prospective memory targets improved the accuracy of prospective memory under motor and verbal encodings. We therefore infer that related association contributes to sememe heredity of action semantics, and the process does not depend on external motor encoding.

## General Discussion

The free recall performance based on the priming phrases in Experiment 1 and the learning phrases in Experiment 2 showed that the enactment effect was significant. This is one of the most reliable findings in research on memory since the early 1980s (Saltz and Donnenwerth-Nolan, 1981; Yu and Wang, 2017). In addition, these experiments revealed new results.

In Experiment 1, a subject-performed task was combined with the priming effect to explore the contribution of verbs and nouns in action phrases from the encoding stage directly. The results showed that verb-specific processing markedly contributed to the enactment effect. This study expands the scope of previous studies which only verified the motor encoding theory from the perspective of memory retrieval (e.g., Engelkamp and Zimmer, 1994; Steffens et al., 2006). The five-component view of the SPT task only proposed that motor encoding makes individuals pay more attention to the action components of phrases (Zimmer, 2001). This viewpoint has been validated in this study for the first time.

However, our results do not support the episodic integration theory. This is because the action phrases used in this study were well collocated, hence the contribution of the integration between verbs and nouns to the enactment effect was not prominent (Kormi-Nouri, 1995; Kormi-Nouri and Nilsson, 2001). The five-component theory of SPT tasks states that lexical-semantic processing is the first stage of motor encoding in which the motor components of materials are focused so that relevant action representations and motor-semantic representations are activated (Zimmer, 2001). As a result, verb semantics are likely to be handled preferentially in action semantic processing.

The related association of action semantics treats action semantic as a sememe unit, and the sememe heredity of action semantics can be derived from verb sememe heredity or noun sememe heredity. The inferential inheritance of verb semantics includes degree type, result type, degree + result type, and general inference. These sememe heredities are completed by relevance, association, and inference of different verb sememes (Su, 2003). The above analysis reveals that verb-semantic association plays a greater role than noun-semantic association in the sememe heredity of action semantics. In the sememe heredity of noun semantics (Zhang, 1992; Tan, 2018), the association between nouns is simple to understand (Earles and Kersten, 2000). The generation of new meanings can be considered a process of creative interpretation, and similarity association is probably the basis of creative interpretation (Lv and Dai, 2000).

The results obtained from Experiment 1 illustrates that verb-semantic association may play a greater role in the sememe heredity of action semantics than noun-semantic association. Although the determination of hereditary sememes is complicated, it can be roughly divided into four steps: (1) State the main meaning of the word. (2) Determine the original meaning. (3) Complete a sememe analysis of the listed meanings, item by item. (4) Determine the hereditary sememes and illustrate the extended sequence of word meanings. The last two steps are more difficult than the rest (Zhang, 1992). The association between verbs is also more difficult to determine (Earles and Kersten, 2000), making it difficult to determine the hereditary sememes of action semantics. This study reveals that when analyzing the semantics of actions item by item, we should pay more attention to the meaning analysis of verbs. Su (2003) stated that methods used to perform semantic analysis for verbs include transforming the original semantics, adding new semantics, intercepting part of semantics, replacing key semantics, inferencing related semantics, and making comprehensive semantic analysis.

In Experiment 2, we combined a subject-performed-task with a dual task. This differs from previous studies in which only the nouns of action phrases in an ongoing task were semantically assessed (Passolunghi et al., 1995; Pereira et al., 2012a, b; Li and Wang, 2015). The results of Experiment 2 showed that prospective-memory accuracy lacked a significant enactment effect, confirming that this effect is independent of the relational processing between action phrases. Moreover, the category relation of nouns in action phrases (Golly-Häring and Engelkamp, 2003; von Essen, 2005), the relation between item and environment (Helstrup, 1996), as well as the relation process of repeated learning (Engelkamp and Zimmer, 2002; Koriat and Pearlman-Avnion, 2003) did not contribute to the enactment effect.

Analysis of the results also showed that accuracy of prospective memory targets related to learning phrases was significantly higher than that of prospective memory targets unrelated to learning phrases. Notably, this difference was more pronounced under the motor than the verbal encoding condition. We therefore infer that the relational processing between action phrases contributes to sememe heredity, and that sememe heredity is not influenced by external motor encoding. In other words, the sememe heredity of action semantics is based mainly on the semantic content rather than the semantic form (such as external motor encoding of action phrases). This conclusion is in line with the results of action memory. To be specific, external motor encoding and internal imaging-performance encoding have a shared process, which includes movement, dynamic motion, and visual information (Leynes and Kakadia, 2013). Besides, there is no significant difference in cued recall (Engelkamp, 1995) or activation of the motor cortex (Senkfor, 2008) between the above two encoding conditions. In comparison with verbal retrieval, motor retrieval did not improve recall performance significantly (Engelkamp, 2001).

This study conducted empirical data analysis on the sememe heredity of action semantics. However, the results of this study should be interpreted with caution because action memory is only an expression of the sememe heredity of action semantics, not its entirety. Future studies should aim to obtain multi-dimensional and comprehensive data about sememe heredity of action semantics from the perspectives of object recognition, motion simulation, and so forth.

## Data Availability Statement

The datasets generated for this study are available on request to the corresponding author.

## Ethics Statement

The studies involving human participants were reviewed and approved by the Special Committee on Scientific Research Ethics and Academic Evaluation of Jiangsu Normal University. The patients/participants provided their written informed consent to participate in this study.

## Author Contributions

LW and ZY designed, researched, and wrote the manuscript. YM collected and analyzed the data. All authors contributed to the article and approved the submitted version.

## Conflict of Interest

The authors declare that the research was conducted in the absence of any commercial or financial relationships that could be construed as a potential conflict of interest. The reviewer SZ declared a past collaboration with one of the authors LW.
